# Halogenated Compounds from Marine Algae

**DOI:** 10.3390/md8082301

**Published:** 2010-08-09

**Authors:** Maria Teresa Cabrita, Carlos Vale, Amélia Pilar Rauter

**Affiliations:** 1 IPIMAR, Av. de Brasília, 1449-006 Lisboa, Portugal; E-Mail: cvale@ipimar.pt (C.V.); 2 Centro de Química e Bioquímica/Departamento de Química e Bioquímica da Faculdade de Ciências da Universidade de Lisboa, Ed C8, Piso 5, Campo Grande, 1749-016 Lisboa, Portugal; E-Mail: aprauter@fc.ul.pt (A.P.R.)

**Keywords:** marine algae, halogenated compounds, biotechnological applications, ecological role

## Abstract

Marine algae produce a cocktail of halogenated metabolites with potential commercial value. Structures exhibited by these compounds go from acyclic entities with a linear chain to complex polycyclic molecules. Their medical and pharmaceutical application has been investigated for a few decades, however other properties, such as antifouling, are not to be discarded. Many compounds were discovered in the last years, although the need for new drugs keeps this field open as many algal species are poorly screened. The ecological role of marine algal halogenated metabolites has somehow been overlooked. This new research field will provide valuable and novel insight into the marine ecosystem dynamics as well as a new approach to comprehending biodiversity. Furthermore, understanding interactions between halogenated compound production by algae and the environment, including anthropogenic or global climate changes, is a challenging target for the coming years. Research of halogenated metabolites has been more focused on macroalgae than on phytoplankton. However, phytoplankton could be a very promising material since it is the base of the marine food chain with quick adaptation to environmental changes, which undoubtedly has consequences on secondary metabolism. This paper reviews recent progress on this field and presents trends on the role of marine algae as producers of halogenated compounds.

## 1. Introduction

Marine algae produce a wide variety of remarkable natural compounds, usually referred to as secondary metabolites because they are not involved in the basic machinery of life [1]. Although these molecules often contribute to only a very small fraction of the organism total biomass [2], the contribution of these compounds to survival may sometimes be comparable to metabolites resulting from the primary metabolism [3]. In that sense, the use of the term “secondary metabolite” seems less appropriate since these compounds also contribute to growth, reproduction and defense and thus play a primary role for the organism integrity.

Many of these secondary metabolites are halogenated, reflecting the availability of chloride and bromide ions in seawater. Interestingly, bromide is more frequently used by algae for organohalogen production, although chlorine occurs in higher concentrations than bromine in seawater. Marine halogenated compounds comprise a varied assembly of compounds, ranging from peptides, polyketides, indoles, terpenes, acetogenins and phenols to volatile halogenated hydrocarbons [4]. The prevalence of halogens is not similar in marine algae: chlorine and bromine appear to be the main halogens used to increase biological activity of secondary metabolites, whereas iodine and fluorine remain quite unusual within the chemical structures [5]. However, some orders of brown algae such as Laminariales accumulate and use iodine for halogenation processes. For example, the kelp *Laminaria digitata* accumulates iodine to more than 30,000-times the concentration found in seawater, representing an average content of 1% of dry weight [6]. In fact, iodination is more frequent in brown algae than in red and green algae metabolites [6]. As a result, only less than 1% of secondary metabolites from of brown algae contain bromine or chlorine in contrast with as much as 90 and 7% of red and green algal compounds, respectively [7].

Halogenation often provides these compounds with interesting key features and marine algae hold diverse and unique biosynthetic pathways for the production of halogenated metabolites. The halogenated sesterterpenes, neomangicols A–C, isolated from the marine fungus *Fusarium* [8], offer a striking example of the halogenation effect. Neomangicol A and B display *in vitro* cytotoxic effect toward the HCT-116 human colon tumor cell line, while their nonhalogenated analog neomangicol C was shown to be inactive [8].

Biological properties of halogenated compounds have been researched for the past decades, with results showing antibacterial, antifungal, antiviral, anti-inflammatory, antiproliferative, antifouling, antifeedant, cytotoxic, ichthyotoxic, and insecticidal activity [9]. Research major focal points indubitably have been the discovery and characterization of new halogenated compounds, along with a remarkable effort toward the evaluation of their possible biomedical and biotechnological applications. Also, chemical profiles have been used to differentiate cryptic species but the chemotaxonomic value of natural halogenated compounds has been questioned due to geographical and seasonal variations in the chemical composition of algal species [10]. The ecological role of marine halogenated metabolites, alongside with other natural compounds, has been disregarded, although a few halogenated metabolites have been shown to have important and critical roles on the community structure of marine ecosystems in previous years [11–17]. In fact, most of the publications on marine ecological issues rarely include a chemical approach of compounds responsible for the ecological interactions found. Excellent reviews on marine natural compounds have been recently published [9,18,19], with no particular emphasis on halogenated metabolites. These showed a variety of structure types that were isolated from a wide range of marine organisms, including microorganisms and phytoplankton, macroalgae, sponges, cnidarians, bryozoans, molluscs, tunicates, echinoderms and true mangrove plants. During 2009, new naturally occurring algal halogenated compounds were found, most of them with little or no reported biological activity. Reports on the ecological role of algal natural compounds, presenting both chemical and ecological approaches were still few. The present paper focuses on halogenated compounds only, and is restricted to micro and macroalgae as natural sources. It attempts to provide an overview of new marine halogenated compounds reported in 2009 and to examine progress and present trends on the role of marine algae as producers of halogenated compounds.

## 2. Macroalgae

Interest in the actual marine natural compounds responsible for such a wide range of properties is far more recent and new compounds are still being discovered and isolated, chemical structures elucidated and properties being sorted out, with the aim of finding new molecular entities with industrial application. From time to time, a new compound emerges as particularly promising in a specific area and additional testing starts to allow further pharmacological evaluation.

Many compounds have been found in marine macroalgae in recent years, mainly in red and brown algae, and fewer in green algae [9]. Among all marine macroalgae, red algae are the main producers of halogenated compounds. *Laurencia* (family Rhodomalaceae, order Ceramiales, class Rhodophyceae, phylum Rhodophyta) is considered one of the most prolific genera [20,21], being mainly found in tropical, subtropical, and temperate coastal waters. It has been intensively screened over the last fifty years, although a variety of new halogenated molecules are still being reported. Characteristically, the halogenation degree found in compounds from *Laurencia* is relatively high [19]. Diterpenes sesquiterpenes, triterpenes, and C15-acetogenins are the main secondary compounds of this genus [22,23] with which antimicrobial [24], antifeedant [25], antihelmintic [26,27] and cytotoxic [28,29] properties are generally associated.

**Figure 1:**
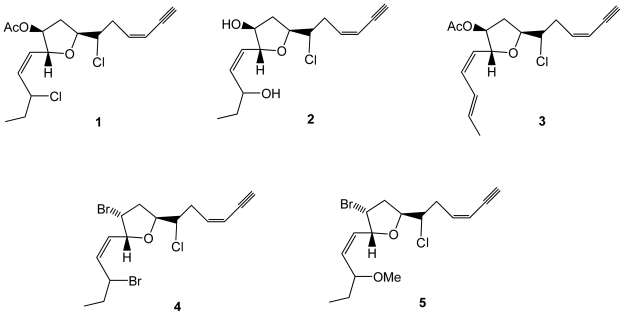
Structures of compounds **1**–**5** isolated from *Laurencia glandulifera*.

Five new chlorinated compounds, C15 acetogenin en-ynes (**1**–**5**), were obtained from *Laurencia glandulifera* collected from the island of Crete (Figure 1). Four of them were evaluated for their cytotoxicity toward HT-29 (colorectal adenocarcinoma), MCF-7 (mammary adenocarcinoma), PC-3 (prostate adenocarcinoma), HeLa (cervical adenocarcinoma), and A431 (epidermoid carcinoma) human tumor cell lines, but no significant activity was found [30]. The characteristic terminal *cis* ene-yne moiety is also present in C15 eight-membered cyclic ethers previously isolated from *L. glandulifera* collected on the Crete Island, which exhibited antistaphylococcal activity with minimum inhibitory concentrations (MICs) in the range of 8–256 mg/mL [31].

**Figure 2:**
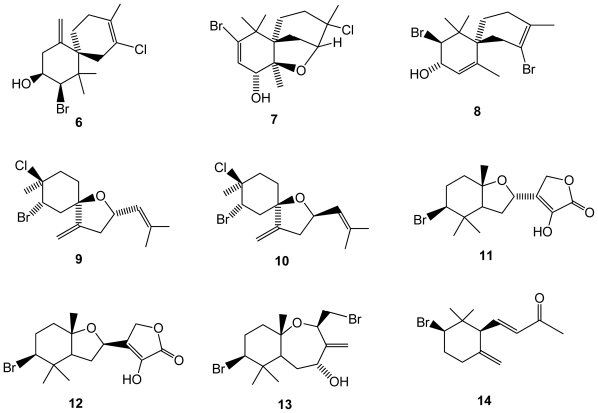
Structures of halogenated compounds **6**–**14** isolated from *Laurencia saitoi*.

Elatol (**6**)(Figure 2), a halogenated sesquiterpene alcohol, commonly found in many species of *Laurencia*, and known for its potent antibacterial activity, was isolated for the first time in *Laurencia microcladia*, collected in the Southern Brazilian coast [32]. Previous analysis of anti-herbivory proprieties of metabolites from *Laurencia* species (including elatol) have been conducted but no investigation had been reported for *Laurencia microcladia*. Elatol anti-herbivory properties were investigated using the black sea urchin *Echinometra lucunter*, but it was found that this species can tolerate elatol, at least in the concentrations tested [32]. However, this compound was able to deter feeding of *Thalassia* by reef fishes with a reduced loss of *Thalassia* by 60% and was shown to be a very efficient deterrent against the sea urchin *Diadema antillarum*, reducing grazing by 86% [11]. However, anti-herbivory proprieties could not be detected by testing the compound on the black sea urchin *Echinometra lucunter*.

*Laurencia saitoi*, with exceptionally few reported halogenated compounds [25] in the past, was found to produce four novel halogenated sesquiterpenes: 10-bromo-3-chloro-2,7-epoxychamigr-9-en- 8-ol (**7**), 2,10-dibromochamigra-2,7-dien-9-ol (**8**), (9*S*)-2-bromo-3-chloro-6,9-epoxybisabola-7(14),10-diene (**9**), and (9*R*)-2-bromo-3-chloro-6,9-epoxybisabola-7(14),10-diene (**10**)[33](Figure 2). In addition, other brominated compounds were already reported for different macroalgal species, namely aplysistatin, a well known antileukemic agent [34,35], 5-acetoxypalisadin B, palisadin A, palisadin B belonging to a series of antimicrobial compounds, and 2,3,5,6-tetrabromoindole [36]. Cytotoxicity of the isolated compounds was evaluated by the MTT method, all the tested compounds being found inactive [36]. New brominated sesquiterpenes and a norsesquiterpene were also reported from the same species [37], namely 2-hydroxyluzofuranone (**11**), 2-hydroxyluzofuranone B (**12**), 4-hydroxypalisadin C (**13**), and 2-bromo-γ-ionone (**14**)(Figure 2).

**Figure 3:**
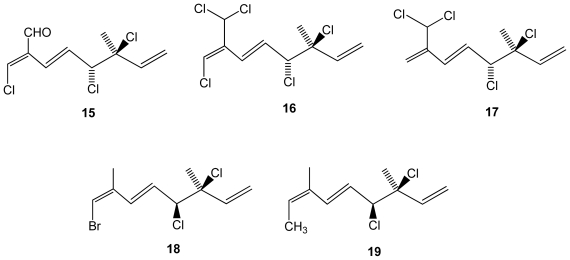
Structures of monoterpenes **15**–**19** isolated from *Plocamium cornutum*.

Other red algal genera have also been screened and interesting findings were recently reported. From the chemical-rich genus *Plocamium*, a South African red algae *Plocamium cornutum* yielded five halogenated monoterpenes (**15**–**19**) from which two are new (compounds **18**–**19**)(Figure 3). The antiplasmodial activity against the chloroquine sensitive strain of the most frequent and deadly human malaria parasite *Plasmodium falciparum* was tested. Although the compounds were significantly less active than standard chloroquine, compounds **16** and **17** containing 7-dichloromethyl moiety were the most active ones (IC_50_ = 16 and 17 μM, respectively), followed by compound **15** (IC_50_ = 27 Mm), which contains an aldehyde functional group at this position, while the new compounds were essentially inactive [38].

**Figure 4:**
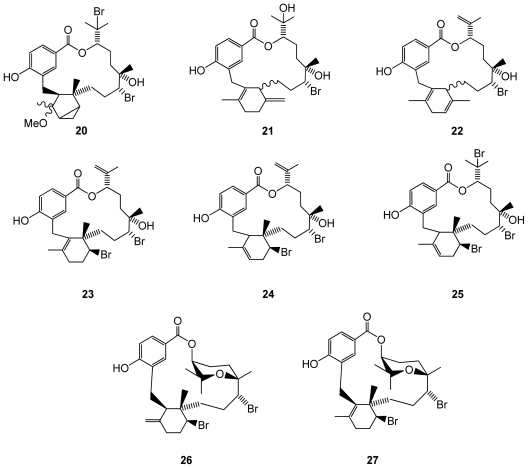
Structures of bromophycolides **20**–**27** from *Callophycus serratus*.

Another red algal species, the Fijian *Callophycus serratus*, was found to produce eight bromophycolides J–Q (**20**–**27**)(Figure 4), which exhibited IC_50_ values against *Plasmodium falciparum* in the low micromolar range (44 for compound **22**, 0.5 for **23**, 1.4 for **24**, **25** and **27**)[39]. Antimalarian activity appeared to be associated with the presence of a macrolide motif in the chemical structure of these molecules. Antibacterial assays were performed using methicillin-resistant *Staphylococcus aureus* and vancoymcin-resistant *Enterococcus faecium* as test pathogens. The macrolides were also evaluated against a panel of 12 tumor cell lines including breast, colon, lung, prostate, and ovarian cancer cells. Of the eight tested compounds, bromophycolides **26** and **27** exhibited the most potent antibacterial activity against *S. aureus* and *E. faecium,* suggesting that conformational rigidity and/or hydrophobicity conferred by the tetrahydropyran system contributes to antibacterial activity. While all tested bromophycolides exhibited moderate antineoplastic activity, only **24** displayed some cell line selectivity, with an IC_50_ of 1.5 μM against the breast tumor cell line. Interestingly, while **24** demonstrated cancer cell line selectivity, its regioisomer **23** was quite active against all cancer cell lines tested (IC_50_’s 2.1–7.2 μM). Bromophycolide **27** was the most potent *C. serratus* natural product evaluated but showed little cell line selectivity.

**Figure 5:**
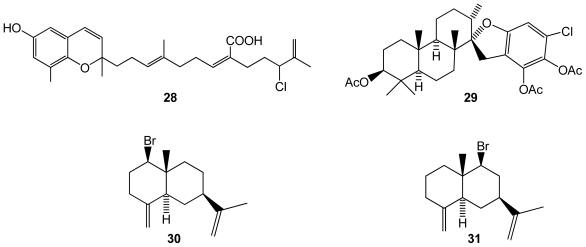
Structures of compounds **28**–**31** isolated from brown algae.

Halogenated metabolites from brown algae, reported as unusual for this algal group [40], have been found to be mainly terpenes. However, some new molecules were reported for brown algae in 2009. A new halogenated meroditerpenoid, fallachromenoic acid (**28**)(Figure 5), was isolated from the southern Australian brown algae *Sargassum fallax*, and displayed antitumor activities against a P388 Murine Leukaemia cell line [41]. Although there were no previous reports of halogenated compounds in the *Stypopodium* genus, Areche *et al.* [40] found an unusual 40-chlorostypotriol triacetate (**29**)(Figure 5) in *Stypopodium flabelliforme*, from which biological properties are still unknown. Another brown algal species, *Dictyopteris divaricata*, was found to produce two new brominated selinane sesquiterpenes, 1-bromoselin-4(14),11-diene (**30**) and 9-bromoselin-4(14),11-diene (**31**)[42](Figure 5).

No reports associated with green algae were found in the selected bibliography from 2009.

## 3. Cyanobacteria

Marine phytoplankton has been much less investigated than macroalgae although marine microorganisms are, in general, increasingly considered successful sources of natural products. Earlier reviews and reports on marine natural products including phytoplankton refer mainly to cyanobacteria and dinoflagellates [9,18,43,44] as sources of natural compounds.

Selected examples of recently discovered halogenated molecules produced by marine phytoplankton, from 2009 literature, exhibited the same trend. Among phytoplankton major groups, cyanobacteria have come forward as one of the most promising groups of microorganisms for the isolation of interesting new natural halogenated compounds [45,46]. A wide range of compounds with different properties is produced by these blue-green algae [44]. For example, many of these compounds were found to be anticancer agents or powerful neurotoxins performing either as blockers or activators of the eukaryotic voltage-gated sodium (Nav) channels [44].

Cyanobacterial toxins have received increased research effort in recent years [47–49] because these molecules are among the most found hazardous substances in surface waters and occurrence of harmful cyanobacterial bloom events is rising worldwide [50].

**Figure 6:**
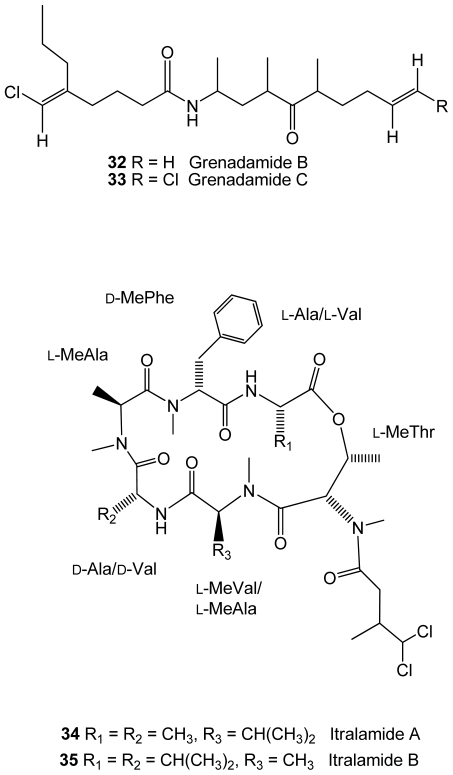
Structures of compounds **32**–**35** isolated from the cyanobacteria *Lyngbya majuscula*.

The cyanobacteria *Lyngbya majuscula*, from an Eastern Caribbean collection, was found to produce two new halogenated fatty acid amides, grenadamides B and C (**32**–**33**), and two new depsipeptides, itralamides A and B (**34**–**35**)[51](Figure 6). The first two compounds displayed marginal activity against the beet armyworm (*Spodoptera exigua*).

**Figure 7:**
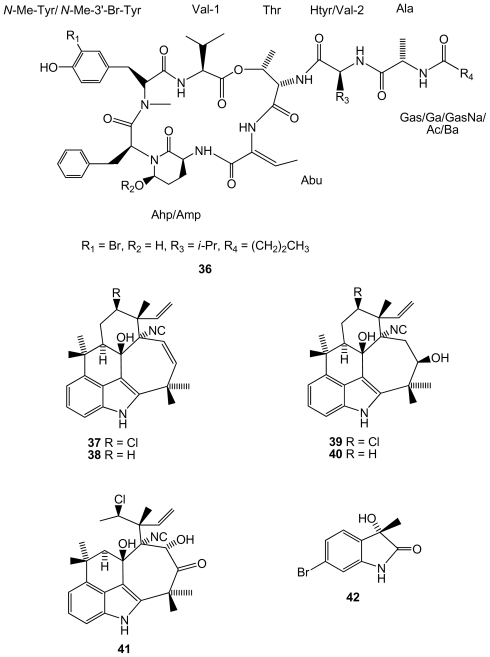
Structures of compounds **36**–**42** isolated from cyanobacteria.

From the same genus, a new brominated compound, lyngbyastatin 10 (**36**)(Figure 7), closely related to lyngbyastatins, was found in *Lyngbya semiplena*, collected in Tumon Bay (Guam, Pacific ocean), inhibiting porcine pancreatic elastase [52]. Another cyanobacteria, *Fischerella ambigua*, obtained from a culture, produces five newly found antibacterial chlorinated molecules, ambiguine K–O isonitriles (**37**–**41**)[53](Figure 7). Ambiguine K and M isonitriles showed the most potent activity against *Mycobacterium tuberculosis*, the causal agent of most cases of tuberculosis. Ambiguine A isonitrile was the most active one against *Bacillus anthracis*, the bacterium that causes anthrax. A new brominated indole alkaloid, named as bromoanaindolone, (IUPAC name: 6-bromo-3-hydroxy-3-methyl-indol-2-one)(**42**)(Figure 7), yielded by the cyanobacteria *Anabaena constricta* [54], showed antibacterial activity against the Gram-positive species *Bacillus cereus*, the bacterium that causes foodborne disease commonly known as food poisoning. Bromoanaindolone also displayed anticyanobacterial activity against the filamentous species *Arthrospira laxissima* and *Nostoc carneum*, and also against the unicellular species *Chroococcus minutus*, *Synechocystis aquatilis* and *Synechococcus* sp., which indicates the allelopathic potential of this compound.

## 4. Limitations and Possible Solutions to Detection of Bioactivity

The fact that some of the newly found halogenated compounds show minor or no activity at all against a specific target does not exclude the possibility of other hidden unidentified active biological effects. Additionally, the supposedly absence of bioresponse may be related to limitations in the analytical chemistry methods employed. Bioassay-guided fractionation is the iterative approach traditionally applied to disclose bioactive natural compounds [55,56]. Difficulties in perception of bioactivity may be linked to compound instability, caused by fractionation and isolation from the natural cellular environment or simply because compounds may act synergistically or more than one compound may be associated to a specific property. This is particularly true for macroalgae and microalgae which are complex compound matrices [57]. Metabolite-oriented approaches, namely metabolite profiling and metabolomics, may supplement bioassay-guided fractionation [58]. These two approaches are focused on the chemical properties of the metabolome, which includes the complete set of metabolites of an organism (metabolomics), or of a limited group of pre-defined metabolites (metabolite profiling)[59]. In the latter approach, pre-defined metabolites are selected either according to the class they belong to (organic phosphates, carbohydrates, *etc.*) or to an associated specific pathway. Both metabolite profiling and metabolomics allow all compounds to be simultaneously measured, so that detection of unstable compounds is facilitated and synergistic effects detected [59]. Also, the influence of a particular condition on the organism physiology can be revealed through qualitative and quantitative differences in the metabolic profiles obtained [60,61]. This could be a valuable tool for the assessment of effects of environmental changes on the community structure and organism physiology. However, useful metabolomics may be used for a more thorough and complete detection of metabolites, limitations subsist when applied to chemical ecology, due to the difficulty in establishing a link between one or more specific metabolites and an ecological role [58].

Other techniques have been applied for the study of halogenated compounds in marine algae. Desorption electrospray ionization mass spectrometry (DESI-MS) and some applications, such as imaging, have very recently started to be used for natural products detection on intact algal tissue surfaces [62,63]. DESI-MS is an ambient ionization technique coupled with mass spectrometry, allowing probing the surface of solids, liquids, frozen solutions, and adsorbed gases [64]. In ambient DESI-MS, samples are kept under atmospheric pressure in open air, with minimum or no preparation at all [64]. Tissue surfaces are subject to a focused spray of charged particles of a polar solvent, which leads to the release of intact gaseous ions from surfaces into the mass spectrometer [65]. Low detection limits and high analysis speed are achieved, and innate chemical information is preserved to a maximum [64]. DESI-MS has been shown to be a sensitive and effective approach to detect algal diterpene-benzoate macrolide natural products, namely bromophycolides, directly on the surface and interior of a marine red alga, *Callophycus serratus* [62]. Reactive DESI approaches were investigated to improve ionization efficiency and detection limits. Different polar solvent compositions, tested directly on the algal surfaces, were demonstrated to enhance sensitivity for discovery of various bromophycolides, which would have otherwise been undetected. Imaging, one of DESI-MS possible applications, has been shown to provide an exceptional capability to map secondary metabolites to distinct algal surface sites [63]. Bromophycolides were shown to be non homogenously distributed across surfaces but instead associated with distinct patches, again in the red algae *Callophycus serratus*. DESI-MS imaging appears to be a powerful tool for the investigation of the function of surface-associated natural products in ecological interactions, as spatially-resolved measurements with lateral resolutions in the hundreds of micrometers are made possible. These studies [62,63] illustrate the potential of DESI-MS and its applications, such as imaging, in understanding chemically-mediated biological processes in macroalgae.

## 5. Ecological Role

Micro and macroalgal survival and success in the marine environment reflect to a great extent the many strategies these organisms have had to employ throughout their evolutionary history to adapt to a hostile and high resource-demanding milieu. Extreme competition for space, light and nutrients has shaped the organisms’ physiology, resulting in a complex array of biologically active metabolites characteristic of each species. Many of these secondary metabolites have been shown to support the defense mechanisms developed by marine algae [66–69]. Secondary metabolites are now suggested to be responsible for marine biodiversity on the genetic, species, and ecosystem levels [70,71].

Chemical ecology, which tackles the role of natural compounds in the interactions between organisms and also between organisms and their environment, has already a few decades of investigation dedicated to marine systems [46]. Historically, interest in natural products from marine algae was initially driven to meet pressing biomedical requirements for new drugs against fungal [72], parasitic [73], bacterial [74], and viral [75] diseases, which were either used in their natural form or as templates for synthesis and further chemical structure modification [46]. For this purpose, macroalgae from tropical areas were heavily screened and many new halogenated compounds were found in the last decade [18]. Apart from potent and original therapeutic agents for infectious diseases and cancer, among many other disorders, natural products isolated from marine algae were also found to clearly control interactions between organisms [11–13], and therefore influence population structure, communities organization, and ecosystem function [14–17]. Biodiversity in marine systems may be a result of chemical diversity [68]. Presently, specific ecological aspects such as chemical sensing of the environment by algae, intraspecific signaling, allelopathy, predator-prey and host-parasite interactions, and bioaccumulation and transfer of toxins within food webs have been addressed on recent research on the ecology of natural compounds from marine algae. Excellent reviews and essays on marine chemical ecology covering years prior to 2009 have been published (see [3,17,68,76–78]), however, with no particular focus on halogenated compounds. Interactions between algae and herbivorous invertebrates or describing allelopathic effects have received most of the attention. These ecological interactions continue to be predominantly documented from an ecological perspective [79,80]; the chemical identification of compounds responsible for the found ecological relationships was overlooked during 2009. A multidisciplinary approach including organic chemistry, biology, and ecology components is required for the in depth elucidation of the ecological relationships in the near future. So far, the ecological and the chemical approaches rarely coincide in the same study; the cellular and molecular bases for these interactions remain disregarded. In fact, to the best of our knowledge, an ecological study on marine macroalgae, including elucidation of chemical structure of molecules responsible for ecological interactions, was found during 2009. A direct fine-scale evaluation of bromophycolides and callophycoic acids was conducted on red macroalga *Callophycus serratus* surfaces in order to evaluate whether these natural compounds had a role in surface-mediated defense against pathogenic microbes [63]. These novel compounds had been reported for the first time in *C. serratus* [39], represent the largest group of algal antifungal compounds described to date, and were shown to inhibit growth of *Lindra thalassiae*, a marine pathogenic fungus [63]. DESI-MS imaging revealed bromophycolides among heterogeneous patches on algal surfaces and within internal algal tissues. The bromophycolide concentrations found on these surface patches were sufficient suppress growth of *L. thalassia*, indicating that probably these compounds are kept internally and released at sparsely distributed surface sites. The above cited study [63] provides the first direct evidence for localization of chemical signals with spatial resolution <200 μm on biological surfaces in concentrations sufficient for targeted antimicrobial defense, highlighting the potential of DESI-MS imaging for the understanding of small-scale ecological interactions.

## 6. Present Trends

A lot still remains to be done concerning research on the role of marine natural halogenated compounds: (i) isolation and characterization of new halogenated compounds (ii) screening untested micro and macroalgal species (iii) improving assessment in the detection of bioactivities, fully exploring potential of metabolite-oriented approaches, (iv) testing biomedical and ecological relevance of novel and known chemical natural compounds, (v) ensuring reliable taxonomic identification of investigated species, (vi) biogeographical assessing of micro- and macroalgae with relevant compounds, and (vii) developing appropriate strategies for data management.

From a purely ecological point of view, success toward the in-depth understanding of marine ecological interactions is very much dependent on a multidisciplinary approach, relying on adequate bioactivity detection methodologies. Chemical metabolite-oriented approaches may prove to be reliable tools helping to elucidate compound’s biological properties, which may not be detectable in any other way. Ecological studies may just benefit from an interactive and close combination of chemical methodologies with ecological experiments.
